# 

**Published:** 1845-06

**Authors:** 


### WESTERN JOURNAL

OF

### MEDICINE AND SURGERY.

### EDITORS.

---

DANIEL DRAKE, M. D.,

PROFESSOR OF PATHOLOGY AND THE PRACTICE OF MEDICINE IN THE MEDICAL INSTITUTE OF LOUISVILLE:

LUNSFORD P. YANDELL, M. D.,

PROFESSOR OF CHEMISTRY AND PHARMACY IN THE SAME:

THOMAS W. COLESCOTT, M. D.

### INDEX TO VOL. III.

[NEW SERIES.]

A

Abercrombie, death of, 175.
Adhesive plaster, 82.
Administering medicines, most efficient mode of, 258.
Albumen as an antidote, 64.
Alcoholic odor of fluids of the brain, 65.
Animal body, origin of fat in, 341.
Analysis and properties of gastric fluid, 439.
Animal heat, 436.
Antidote, caution in giving albumen as, 64.
Ammonia, decomposition of tinct. opii. by, 62.
Ammonia succinate of, 178.
Amputation in the prairies, 262.
Anatomy and diseases of the urinary and sexual organs, 513.
Arsenic, accidental poisoning with, 156.
Arsenic in carious teeth, 266.
Artificial dilatation of os uteri, 359.
Asafœtida, a preventive of measles, 455.
Ashwell's treatise on diseases peculiar to women, 501.
Asthma, iodide of potassium in, 260.
Asylum, insane, Bloomingdale, 276.
Australia, poison of snakes in, 531.
Avent's case, foreign body in stomach, 198.

B

Baldwin on mustard poultices, 1.
Bampfield on the spine, 233.
Baron Larrey's medical campaigns, 349.
Bartlett on philosophy of medical science, 400.
Belgium, treatment of itch in, 357.
Bell's lectures, 59.
Belladonna in hooping-cough, 446.
Blood, color of, 433.
Blindness, case of, 346.
Bloomingdale asylum for the insane 276.
Blisters, improved mode of managing, 167.
Boardman's discourse, 47.
Boling on the importance of health in clergymen, 283.
Botany, study of, by young physicians, 269.
Breasts, enlargement of, in a male, 452.
Brigham on mental cultivation, 314
British, foreign and medical Review, 365.
Bronchocele, 83.
Buck on diseases of the chest, 277, 400.
Buck on physical diagnosis, 369.
Burgess on calomel in large doses in typhus fever, 161.

C

Calculation, organ of, 81.
Calomel, large doses in typhus fever, 161.
Campbell on Gonorrhœa, 153.
Canada, medical charity fund of, 84
Carr on mercury in southern fevers, 379.
Castor seeds, 82.
Catheterism, best method of performing, 348.
Chancre, prognosis of, 448.
Chapman's Lectures, 24.

Chapman's list of Florida plants, 461.
Christison on poisons, 366, 413.
Charges for homœopathic services, 168.
Cincinnati Journal of Health, 273.
Claims of religion upon medical men, 47.
Color of the blood, 433.
Commencement in medical institute, 362.
Cold affusions in typhus fever, 161.
Concentrated blow-pipe and furnace, Somerby's, 363.
Congestive fever, 179.
Constipation, 74.
Cooper on the testis, 275.
Creation, physical position of man in, 441.
Croton oil in cure of nævi, 172.
Curvature and diseases of the spine, essay on, 233.
Cyclopædia of practical medicine, 58.

D

Dawson's account of putrid sore throat, 93.
Death of Dr. Abercrombie, 175.
Decomposition of tinct. opii, 62.
Delirium tremens, succinate of ammonia in, 178.
Dictionary of practical medicine, 548.
Digestive properties of gastric fluid, 439.
Disease of ovaries, 83.
Diseases of negro population, 164.
Diseases of the skin, 238.
Diseases of the urinary and sexual organs, 513.
Diseases of women, Ashwell's treatise on, 501.
Diseases of the chest, lectures on, 277, 484.
Diseased kidney, case of, 383.
Divided nerve, reunion of, 83.
Drake on diseases of negro population, 164.
Daniell, Professor, death of, 532.

E

Early puberty in Greece, 81.
Education of the blind, Kentucky institution for, 274.
Elements of materia medica, by Harrison, 219.
Emphysema, case of, 11.
Empyema, case of, 386.
Engrafting of nerves, 449.
Epidemic erysipelas, 180.
Erysipelatous fever, observations on 12.
Essay on curvatures and diseases of the spine, 233.
Esquirol on insanity, 546.
Ethics of medicine, 47.
Exemption of Cherokee indians and Africans from insanity, 449.
Examiner, Medical, 180.
Extirpation of uterus for complete inversion, 168.

F

Fat, origin of, in animal body, 341.
Festival of the potato, 444.
Fevers, intermittent, remittent, and congestive, 179.
Florida plants, list of, 461.
Fluid, alcoholic, in brain, 65.
Forensic medicine, 519.

G

Galvanism in growing potatoes, 531.
Gases, liquefaction of, 528.
Gastric fluid, analysis and properties of, 439.
Genital organs, atrophy of, 452.
Geology, lectures on, 361;
Gonorrhœa, abortive and curative treatment of, 153.
Gonorrhœa, 342.
Greece, early puberty in, 81.
Gums, scarification of, 63.
Guthrie on diseases of the urinary and sexual organs, 513.
Guy's principles of forensic medicine, 519.

H

Hamburg, Jewish physicians in, 451.
Harris' case of Emphysema, 11.
Harrison's materia medica, 219.
Health, Cincinnati Journal of, 273.
Health, influence of mental cultivation and excitement upon, 314.
Hemorrhage, uterine, galvanism in, 527.
Hernia, 530.
Home, 445.

Homœopathic services, charge for, 168.
Hooping-cough, treatment and effect of, 446.
Horner's medical topography, 201.
Hydrated peroxide of iron, antidote for arsenic, 156.
Hydrocele, treatment of, 175.
Hydrocyanic acid, poisoning by, 524.
Hydrocele, cure of, 526.
Hygienic condition of towns, 69.

I

Improved mode of managing blisters, 167.
Importance of health in clergymen, 283.
Intermittent fever, 179.
Insane, Bloomingdale asylum for, 276.
Influence of mental cultivation on health, 314.
Insane, proportion of, 445.
Insanity, exemption of Cherokee indians and Africans from, 449.
Insanity, incubation of, 450.
Insanity, Esquirol on, 546.
Introductory lecture, Paine's, 367.
Introductory lecture, 88.
Institute, medical, Louisville, 83.
Ioduretted injections in hydrocele, 175.
Iodide of potassium in asthma, 260
Iodide of iron, pills of, 357.
Iron, hydrated peroxide of, 156.
Itch, treatment of, in Belgium, 357.
Kentucky institution for the education of the blind, 274.
Lunatic asylum, Worcester, 548.

M

Man, physical position of, in creation, 441.
Malpractice, 65.
Magnesia, 339.
Mania a potu, 80.
Materia medica and therapeutics, 219.
Medical men, claims of religion upon, 47.
Medicine, dictionary of, 548.
Medicine, ethics of, 47.
Medicine practical, cyclopædia of, 58
Medical society, Rutherford county, 87.
Medical Society of Tennessee, 545.
Medical Examiner, 180.
Medical topography, Brazil and Uruguay, 201.
Medicines, modes of administering, 258.
Mesmerism, 263, 362.
Medical jurisprudence, Taylor's. 276.
Medical jurisprudence, Guy's 519.
Medical society, London, 342.
Medical practitioners, Paris, statistics of, 347.
Medical campaigne, 349.
Medicine, N. York journal of, 364.
Medical classes, 367.
Medicine, Louisville summer school of, 368.
Medical and surgical journal, St. Louis, 365.
Mercury in southern fevers, 379.
Medical men, longevity of, 453.
Measles, asafœtida a preventive of, 455.
Midwifery, malpractice in, 529.
Mitchell's case of scarlatina, 173.
Miller's principles of surgery, 330.

N

Nævi, cure of, by croton oil, 172.
Nerves, engrafting of, 449.
Negro population, diseases of, 164.
New works, 547.
New medical journal, 547.
Nitrate of silver in gonorrhœa, 153.

O

Ohio lunatic asylum, 163.
Organ of calculation, 81.
Origin of fat in animal body, 341.
Os uteri, dilatation of, 359.
Observations on the botany of Illinois, 187.
Ovarian disease, 83.

P

Paine's introductory lecture, 366.
Pearl's Cæsarian operation, 429.
Phlegmasia dolens, 342.
Physical diagnosis, 277, 369, 485.
Philosophy of medical science, 400
Physical position of man, 441.
Pills, iodide of iron, 357.
Pleurisy, treatment of, 172.
Poisons, treatise on, 366, 413.
Potato, introduction of, 444.
Potatoes, galvanism in growth of, 531.

Pulmonary hepatization, digitalis in, 526.
Practical medicine, cyclopædia of,58.
Practical medicine, dictionary of, 548
Prestat's adhesive plaster, 82.
Prairies, amputation in, 262.
Principles of surgery, 330.
Principles of forensic medicine, 519
Proportion of insane, in the sexes, 445.
Prognosis of chancre, 448.
Prize essays, 184.
Putrid sore throat, 93.
Puberty, Raciborski on, 442.
Punishment of quacks, 268.
Penitentiary, Kentucky, report of physicians of, 92.
Plants of Florida, 461.

Q

Quacks, punishment of, in olden times, 268.
Quack advertisements, 364.
Question of legitimacy, 457.
Question of purity of blood, 485.

R

Raciborski on puberty, 442.
Religion, claims of, upon physicians, 47.
Reasons for scarification of the gums; 63.
Report of physicians to Ky. penitentiary, 92.
Report of cases of scarlatina, 173.
Remittent fever, 179.
Review, British and foreign medical, 365.
Re-union of a divided nerve, 83.
Rheumatism, 337, 342, 522.
Robertson's management of blisters, 167.
Rupture of uterus and abdominal parietes, 429.
Rutherford co. medical society, 87.
Rogers, trial of, 245.

S

Scarification of gums, 63.
Shanks on erysipelatous fever, 12.
Short on the botany of Illinois, 187.
Snakes, poison of, in Australia, 521.
Senecio aureus, 521,
Sneed's case of diseased kidney, 383.
Sneed's case of empyema, 386.
Squill, 358.
Statistics of medical practitioners of Paris, 347.
St. Louis medical and surgical journal, 365.
Southern fevers, mercury in, 379.
Summer school of medicine, 92.
Southern medical and surgical journal, 91.
Succinate of ammonia in delirium tremens, 178.
Students of medical institute, Louisville, mortality among, 181.
Surgery, principles of, 330.
Somerby's blow-pipe and furnace, 363.
Sudden blindness, cure of, 346.
Supporting life on vegetable diet, 68.
Skin, diseases of, 238.
Stackhouse's case of questionable blood, 485.
Surgery, modern improvements in, 534.

T

Taylor's medical jurisprudence, 276.
Testis, Cooper on, 275.
Tinct. opii, decomposition of, 61.
Towns, hygienic condition of, 69.
Tracheotomy, 525.
Treatise on diseases of women, 501
Twins, case of, 527.
Typhus fever, calomel in, 161.

U

Uterus, extirpation of, 168.
Uterine hemorrhage, galvanism in, 527.

W

Worcester on diseases of the skin, 238.
Worcester lunatic asylum, 548.

### *Receipts of Medical Journal for May* 1845.

| | |
|---|---|
| Dr. J. W. Richardson, Stewartsboro, Tenn., | $15 00 |
| Dr. J. Moore, Unionville, Tenn., | 5 00 |
| Dr. J. Morrison, Arcadia, Ill., | 5 00 |
| Dr. Dodd, Bolivar, Miss., | 5 00 |
| Dr. Thos. Sneed, Alexandria, Tenn., | 5 00 |
| Dr. T. J. Todd, St. Joseph's, Mo., | 5 00 |
| Dr. L. F. Robb, Lebanon, Tenn., | 5 00 |
| Dr. T. B. Albertson, Darlington, Ia., | 5 00 |
| Dr. J. R. Moore, county, | 5 00 |
| Dr. V. T. West, Union, Ia., | 10 00 |
| Dr. E. L. Miner, Lethopolis, Ohio, (postage 37,) | 5 00 |
| Dr. Morrow, Savannah, Tenn., | 10 00 |
| Dr. W. D. Harris, Utica, Miss., | 4 84 |
| Dr. S. Slaughter, Cheneyville, La., | 7 84 |
| Dr. J. H. Rodman, Hodgenville, Ky., | 5 00 |
| Dr. J. Cobb, Cumberland Iron Works, Tenn., | 10 00 |
| Dr. R. W. Safford, Pleasant Hill, Ala., | 5 00 |
| Dr. P. H. Lewis, Mobile, | 5 00 |
| Drs. Inge & Calloway, Rockcastle, Ky., | 10 00 |
| Dr. E. B. Haskins, Clarksville, Tenn., | 5 00 |
| Dr. H. Williams, Red Mound, Tenn., | 5 00 |
| Dr. L. S. Tolson, California, Miss., | 5 00 |
| Dr. A. T. Boyd, Begbyville, Tenn., | 5 00 |
| Dr. W. A. Morris, Lynchburg, Tenn, | 10 00 |
| Dr. O. Knox, Waynesboro., | 5 00 |
| Dr. J. B. Hinson, Trenton, Tenn. | 15 00 |
| Dr. J. S. Buford, Whitesville, Tenn., | 5 00 |
| Dr. M. W. Armstrong, Milton, Tenn., | 5 00 |
| Dr. W. Morgan, Germontown, Tenn., | 20 00 |
| Dr. J. A. Green, Covington, Tenn., | 5 00 |

### TO SUBSCRIBERS.

We regret to find that, notwithstanding unusual expenditures upon the present volume of this Journal and its very material improvement, our subscribers have this year been more than usually remiss. We would give notice that all who may not remit promptly shall be forthwith and constantly harrassed with every form of dun until they are worried out. Their names shall appear in a black list on the back of the Journal, and, simultaneously, they shall be called upon and persecuted by duns of every kind, travelling agents, local agents, constables, sheriffs, and catchpoles. We are shamefully deprived of our dues and we mean to have justice done us.

PRENTICE & WEISSINGER.

☞Receipt by mail, at our risk, under frank of post-master or post-paid.

---

### TRAVELING AGENTS FOR THE WESTERN JOURNAL OF MEDICINE AND SURGERY.

JNO. F. JOHNSTON and JAS. H. STEVENSON, Kentucky—W. H. & W. M. WHEELER, Georgia, N. Carolina, S. Carolina, Alabama, and Arkansas—C. W. JAMES, Ohio, Indiana, and Michigan—GEO. C. BRETT, Tennessee—J. F. EASTON, Illinois and Missouri—WM. H. WELD & SON, Mississippi and Louisiana.

---

The WESTERN JOURNAL OF MEDICINE and SURGERY is published monthly by the undersigned, at the corner of Main and Fifth streets, Louisville, at $5 per annum, payable in advance. Each number contains 96 pages, making two volumes in the year of more than 550 pages.

Contributions to its pages by the Physicians of the Valley of the Mississippi, addressed to the Editors, are respectfully solicited.

Letters on business, to be addressed, postage paid, to the publishers.

☞Postmasters, by the regulations of the Postoffice Department, will frank letters containing subscription money, and all remittances so franked are at the risk of the publishers.

*January*, 1844. PRENTICE & WEISSINGER.

